# The Mulberry *WRKY* Transcription Factor *MaWRKYIIc7* Participates in Regulating Plant Drought Stress Tolerance

**DOI:** 10.3390/ijms26041714

**Published:** 2025-02-17

**Authors:** Xueqiang Su, Manli Zhao, Rong Zhou, Cuimin Xu, Ran Zhang, Ruixue Li, Taichu Wang

**Affiliations:** Sericultural Research Institute, Anhui Academy of Agricultural Sciences, Hefei 230000, China; suxueqiangaaas@163.com (X.S.); 13645657446@163.com (M.Z.); mushroomrz@163.com (R.Z.); xu_cuimin2022@163.com (C.X.); 18326199114@163.com (R.Z.)

**Keywords:** mulberry, WRKY TFs, drought stress, transcriptional regulation

## Abstract

The sericulture industry is an important component of the agricultural industry. Drought stress can cause yellowing, premature ageing, and the shrinkage of mulberry (*Morus alba* L.) leaves, greatly damaging their quality and restricting the high-quality development of the sericulture industry. *WRKY* transcription factors play important roles in the plant drought stress response. In this study, we found that *MaWRKYIIc7* of the mulberry *WRKY* TFs, had significantly higher expression levels in leaves than in other tissues and was induced to be expressed under drought stress. The *MaWRKYIIc7* protein is located in the nucleus and plasma membrane, and its transcriptional activity depends mainly on the N-terminal sequence. The overexpression of *MaWRKYIIc7* in *Arabidopsis* resulted in better drought tolerance. An analysis of the transient overexpression of *MaWRKYIIc7* in mulberry seedlings under drought stress revealed that the transgenic seedlings presented decreased stomatal opening, decreased MDA content, increased ROS clearance ability, and increased the expression of ABA biosynthesis-related genes. The Y1H and Dual-luc results indicate that *MaWRKYIIc7* can bind W-boxes to positively regulate *MaNCED1* and *MaRD29A*, synergistically regulating the drought tolerance of mulberry. Overall, our research suggests that *MaWRKYIIc7* can increase plant drought tolerance by promoting ROS clearance, adjusting stomatal opening, and activating the ABA signalling pathway.

## 1. Introduction

Droughts are one of the greatest threats to crop production, and there is a large area of arid or semiarid farmland worldwide, accounting for approximately 50% of the total farmland area. Crops under drought stress experience an average yield reduction of 48% [1,2]. Drought stress can cause damage to normal physiological processes in plants, leading to adverse reactions such as yellowing, atrophy, and a decreased photosynthetic rate [3,4]. As the main source of food for silkworms and a potential health food for humans, mulberry plants mainly produce leaves, which are relatively sensitive to drought or high temperatures [5,6]. At present, silkworm breeding is developing towards industrialisation. Artificial diets for silkworms, made from fresh mulberry leaves, overcome the seasonal limitations of traditional breeding and provide new opportunities for the development of related industries [7]. However, drought stress can reduce the yield of mulberry leaves per unit area, leading to premature ageing, shrinkage, and insufficient nutrient supply, greatly damaging the quality of mulberry leaves and affecting their direct feeding and artificial feed production. This is very unfavourable for the development of the industry and the expansion of its economic benefits.

Analysing the regulatory genes related to plant drought resistance and selecting new plant varieties with high drought resistance through precision breeding are highly important for effectively reducing yield losses caused by drought and stabilising agricultural income [8,9]. Multiple transcription factors are involved in the plant drought stress response, among which *WRKY* transcription factors (*WRKY* TFs) play important roles [10]. The *WRKY* TFs are a class of functionally diverse plant protein superfamilies that are involved in various biological processes, such as plant growth, embryonic morphogenesis, biosynthesis, and stress response [11]. Typical *WRKY* TFs include the WRKY domain (WRKYGQK motif) located at the N-terminus and two types of zinc finger structures (C-X_4-5_-C-X_22-23_-H-X-H, C_2_H_2_ and C-X_5-8_-C-X_25-28_-H-X_1-2_-C, C_2_HC) [12]. Owing to their different genetic structures, *WRKY* TFs can be divided into three subfamilies: group I contains two WRKY domains and one C_2_H_2_; group II has one WRKY domain and one C_2_H_2_ (which can be further divided into five subtypes); and group III consists of one WRKY domain and one C_2_HC [13]. The *WRKY* TFs can exert regulatory functions by binding to the W-box *cis*-acting elements (C/TTGACT/C) in the downstream gene promoters [14].

Since the first *WRKY* TFs were reported in sweet potato in 1994, research on the *WRKY* TFs has been ongoing [15]. At present, researchers have completed the whole-genome identification of *WRKY* TFs in multiple species, such as *Arabidopsis*, rice, and wheat [11]. Among them, the *WRKY* TFs of *Glycine max*, *Brassica napus* and *Panicum virgatum* are relatively large in scale, with the number of family members ranging from 275 to 296 [12,13]. Multiple studies have shown that *WRKY* TFs play important roles in the plant drought stress response and adaptation. The overexpression of *AtWRKY57* significantly improved the drought tolerance of both transgenic *Arabidopsis* and *Oryza sativa* [16]. The heterologous expression of banana *MaWRKY80* in *Arabidopsis* can lead to a greater survival rate, faster growth rate, and lower malondialdehyde and reactive oxygen species contents in transgenic plants under drought stress. *MaWRKY80* can bind to W-box and regulate ABA biosynthesis by activating the expression of *NCED*, thereby promoting leaf stoma motility and water retention [17]. Sorghum *SbWRKY30* can regulate the expression of the drought stress response-related gene *SbRD19*, thereby increasing plant drought tolerance [18]. Soybean *GmWRKY54* can reduce water loss by regulating the opening and closing of stomata. *GmWRKY54* activates the expression of genes such as *PYL8*, *SRK2A*, *CIPK11*, and *CPK3* by directly binding to the promoter region and exerting its drought stress response-related functions through the ABA and Ca^2+^ signalling pathways [19]. Cotton *GhWRKY59* regulates the expression of *GhDREB2* (dependent on W-box *cis*-acting element) and mediates the independent stress response of abscisic acid (ABA) [20]. In summary, *WRKY* TFs constitute an important class of plant drought stress response regulatory genes that can play crucial roles in the precision breeding of highly drought-tolerant crop species.

Baranwal identified 54 *WRKY* TFs in mulberry and investigated the expression patterns of these *WRKY* TFs [21]. However, the expression of mulberry *WRKY* TFs under drought stress and the biological functions of the family members involved in the drought stress response are still unclear. In this study, we further divided the duration of drought stress in mulberry plants into 0, 3, 5, 8, and 10 days and investigated the changes in the expression levels of *WRKY* TFs in mulberry plants under drought stress and in different tissues. These results suggest that *MaWRKYIIc7* is a potential regulatory factor for drought stress in mulberry. We identified the subcellular localization and transcriptional activation activity of *MaWRKYIIc7*. By investigating the transient overexpression of *MaWRKYIIc7* in mulberry seedlings, including changes in stomatal opening and closing, stress-related physiological and biochemical indicators, ROS clearance, and expression patterns of ABA biosynthesis-related genes, we revealed the biological function of *MaWRKYIIc7* in enhancing the plant response to drought stress. Yeast one-hybrid (Y1H), dual-luciferase (Dual-luc) assay results revealed that *MaWRKYIIc7* can rely on a W-box to bind to *MaNCED1* and *MaRD29A* and can participate in the stress response by activating the expression of these genes. In summary, our results enrich functional research on *WRKY* TFs and lay the foundation for elucidating the molecular mechanisms of the drought stress response in mulberry plants.

## 2. Results

### 2.1. WRKY TF Bioinformatics Analysis

We divided the *WRKY* TFs into three subfamilies according to their classification method: Groups I, II, and III. Group I contains nine members, whereas group II is subdivided into five categories: IIa (3), IIb (7), IIc (13), IId (5), and IIe (8). Group III has nine family members. The number of amino acids (AAs) of the mulberry *WRKY* TFs ranged from 180 to 769, with *MaWRKYIIc6* and *MaWRKYIIb6* having the lowest and highest AA numbers, respectively. The results of the calculation of physicochemical parameters revealed a significant difference in the kDa values of mulberry *WRKY* TFs, with members between 20 and 30 kDa accounting for 38.5% of the total (only *MaWRKYI2* and *MaWRKYIIb6* exceeded 80 kDa) (Table S1).

**Figure 1:**
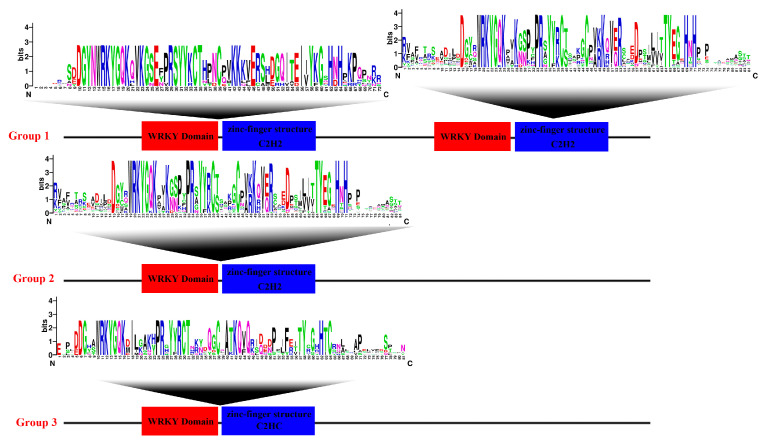
Conserved WRKY and zinc finger domain composition. Alignment analysis of *WRKY* TFs sequences using the ClustalW tool. These domain diagrams were plotted using the online WebLogo tool.

We identified 16 members with pI values greater than 7.5, which were mainly concentrated in the group II subfamily, with a maximum value of 9.68 (*MaWRKYIId4*). The pI values of the nine members of the Group III subfamily are less than 6.5, all of which are acidic amino acids. Only six *WRKY* TFs had a grand average hydropathicity (GRAVY) greater than -1, and the maximum GRAVY value (−0.508) was observed for *MaWRKYIId4*. The subcellular localization prediction results revealed that *MaWRKYIIc4* may be expressed in the chloroplast (chlo) or nucleus (nucl), whereas the remaining *WRKY* TFs were predicted to be expressed in the nucleus. Sequence analysis (Figure 1) revealed that all *WRKY* TFs have typical WRKY domains, and the conservation of the WRKYGQK sequence is high. However, a glutamine mutation at the Q site of *MaWRKYIIc3* and *MaWRKYIIc8* to lysine (K) is the only conserved sequence mutation, indicating the possible functional divergence of these two genes.

### 2.2. Environmental Selection Pressure Analysis of the WRKY TFs

To investigate the main driving forces and environmental selection pressures of *WRKY* TFs, we calculated the Ka, Ks, and Ka/Ks ratios for the 21 duplicated gene pairs in mulberry (Table 1). Ka/Ks > 1 indicates positive selection, whereas Ka/Ks < 1 indicates negative selection [22]. Among the 21 duplicated gene pairs, only *MaWRKYIIe3*-*MaWRKYIIe4* experienced positive selection (Ka/Ks = 1.204), while the Ka/Ks ratios of the remaining duplicated gene pairs did not exceed 0.761, indicating that these duplicated gene pairs experienced strong negative selection. We subsequently collected the *WRKY* TFs sequences of *Arabidopsis*, *Eucalyptus grandis*, *Brachypodium distachyon*, and *Medicago sativa* and compared them with those of mulberry for 4DTV value analysis (Figure S1). Among the five species, WRKY TFs were associated with large-scale duplication events, the peak of 4DTV occurred at values of approximately 0.5.

### 2.3. Analysis of WRKY TFs Expression Patterns

To comprehensively understand the expression specificity of *WRKY* TFs in mulberry, we investigated the changes in the expression patterns of *WRKY* TFs in leaves, fruits, roots, stems, staminate flowers, and pistillate flowers and leaves after drought stress treatment (Figures S2 and S3). The expression levels of *MaWRKYI4* and *MaWRKYIId3* in different tissue parts were relatively low, and *MaWRKYI6*, *IIc1*, *IIc8*, *IId4*, *IId5*, *IIe5*, and *IIe8* were highly expressed mainly in leaves. We identified potential regulatory factors that may be involved in fruit development in various subgroups. These *WRKY* TFs, such as *MaWRKYI5*, *IIa1*, *III1*, and *III4*, contain highly expressed levels in fruits. Most *WRKY* TFs did not have the strongest expression signals in roots or stems, with only *MaWRKYIIc2*, *IId1*, *IId2*, and *III6* (roots) and *MaWRKYIIe3* and *III4* (stems) having higher expression levels than those in other tissues. *MaWRKYI1*, *IIa2*, and *IIe1* presented high expression peaks in staminate or pistillate flowers, which may be involved in the sexual reproduction of mulberry plants (Figure S2).

Mulberry seedlings presented a severe stress phenotype after 10d of drought stress treatment. To fully explain this phenomenon, we conducted in-depth research on the changes in the expression patterns of *WRKY* TFs induced by drought stress (drought stress treatment times were divided into 0, 3, 5, 8, and 10 d) (Figure S3). The results revealed that the transcription of 24 family members (including *MaWRKYI2*, *IIa1*, *IIc4*, *IIc7*, *IIe2*, III8, etc.) was activated by drought stress; the difference was that the expression level of *MaWRKYIIc2* rapidly increased to its highest point at 3 d, the peaks of *MaWRKYIIc7*, *III8* occurred at 8 d, the other *WRKY* TFs in response to drought induction presented the most active transcription at 5 d. Our previous research revealed that *MaWRKYIII8* may play a role as an important regulatory factor in the mulberry drought stress response [23]. The expression pattern of *MaWRKYIIc7* is similar to that of *MaWRKYIII8*, and its expression level is significantly greater in leaves than in other tissues. Therefore, in this study, *MaWRKYIIc7* was used as a potential drought stress regulatory factor to further investigate its biological functions.

### 2.4. Subcellular Localization and Transcription Activity Analysis of MaWRKYIIc7

**Figure 2:**
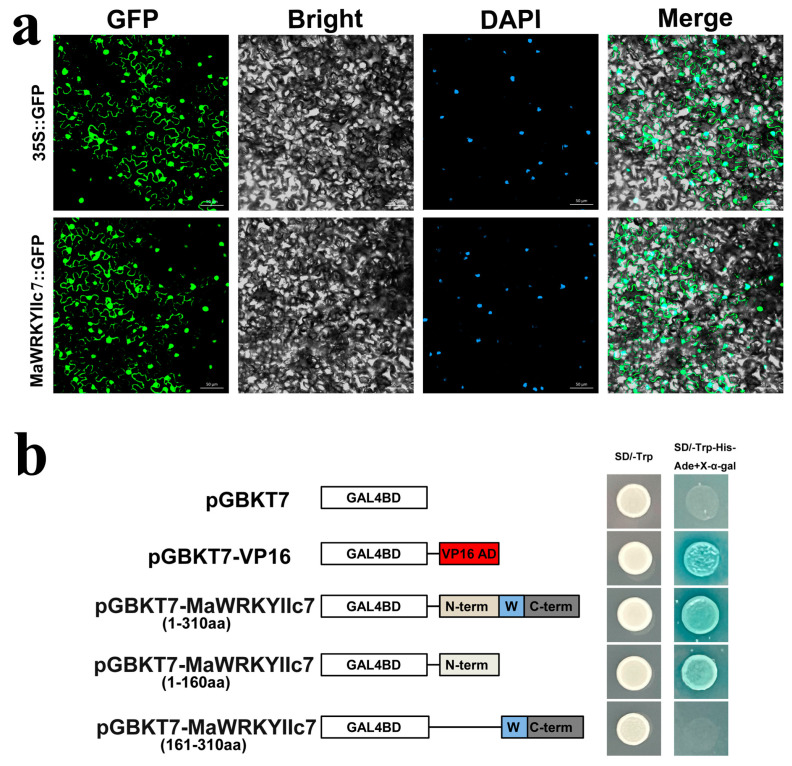
Subcellular localization and transcriptional activation assay of *MaWRKYIIc7*. (**a**) Subcellular localization of *MaWRKYIIc7* in the tobacco epidermis. Fluorescence detection of tobacco leaf epidermal cells, with 35S:GFP transformed tobacco epidermis as control; DAPI is the nuclear localization marker. (**b**) Schematic diagram of different fragments of *MaWRKYIIc7* connected to pGBKT7 vector. Yeast cells are cultured on tryptophan deficient medium (SD-T) and selective medium lacking tryptophan, histidine, and adenine (add X-α-D galactosidase, SD-T/H/A+X-α-gal).

*MaWRKYIIc7* is potentially involved in the response of mulberry to drought stress. We found that some core cis-*elements* in the promoter regions of *MaWRKYIIc7*, such as one MBS (MYB binding site involved in drought stress regulation), CGTCA motif and TGACG motif, are associated with the MeJA response. Notably, the promoter regions contain ABRE, indicating that *MaWRKYIIc7* may also be induced by ABA expression (Table S2). To further clarify the expression patterns of *MaWRKYIIc7*, we constructed a MaWRKYIIc7-eGFP recombinant protein, and green spontaneous fluorescence was detected from both eGFP and MaWRKYIIc7-eGFP via laser confocal microscopy. Transient MaWRKYIIc7-eGFP protein expression was localised in the nucleus and plasma membrane of tobacco leaf cells (Figure 2a).

We attempted to explain the potential transcriptional activity of *MaWRKYIIc7* in the absence of bait proteins. pGBKT7-*MaWRKYIIc7* (1–310 AA, 1–160 AA, and 161–310 AA) was transferred to yeast cells, which were subsequently cultured on SD/Trp-His-Ade+X-α-gal (SD-T/H/A+X-α-gal) plates, together with a pGBKT7 negative control and a pGBKT7-VP16 positive control. The growth of the yeast cells showed that pGBKT7 and pGBKT7-*MaWRKYIIc7* (161–310 AA) could not grow normally on the culture media, but the yeast cells transformed with pGBKT7-VP16 and pGBKT7-*MaWRKYIIc7* (1–310 AA, 1–160 AA) grew well (Figure 2b).

### 2.5. The Overexpression of MaWRKYIIc7 in Arabidopsis Enhances Drought Tolerance

**Figure 3:**
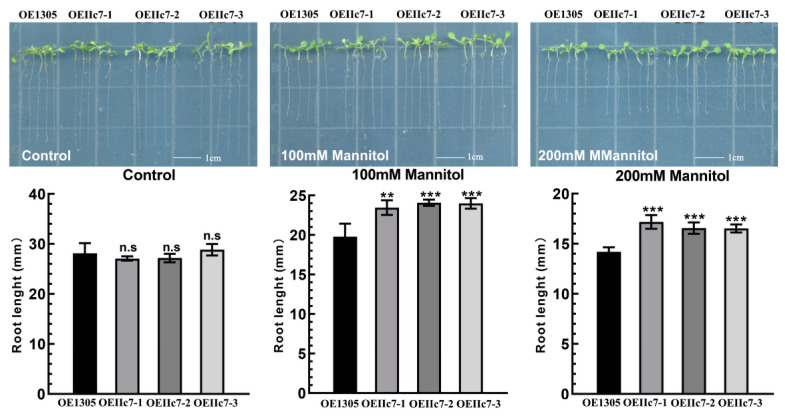
Overexpression of *MaWRKYIIc7* in *Arabidopsis* enhances drought tolerance. Effect of mannitol treatment on *MaWRKYIIc7* overexpression in *Arabidopsis*. Determination of main root length after **5 d** of mannitol treatment (0, 100, and 200 mM). Asterisks indicate the significant difference among OE1305, OEIIc7-1, OEIIc7-2, and OEIIc7-3 lines under the same treatment (** significant difference at *p* < 0.01, *** significant difference at *p* < 0.001, n.s indicates no significant difference).

Through screening with hygromycin plates and qRT-PCR, *MaWRKYIIc7* was identified in 3 lines (OEIIc7-1, -2, and -3); the expression levels of exogenous genes were highest in OEIIc7-2 (the control group was transferred to the pCAMBIA1305 empty carrier, OE1305) (Figure 3 and Figure S4). When mannitol was used to treat transgenic *Arabidopsis* seedlings, the root growth of OEIIc7-1, -2, -3, and OE1305 plants was affected when the concentration of mannitol was 100 mM, but the root length of OEIIc7-1, -2, and -3 plants was greater than that of OE1305 plants. With increasing mannitol concentration (200 mM), the growth of all the transgenic *Arabidopsis* was further inhibited, OE1305 were still more severely affected than the other transgenic *Arabidopsis* (Figure 3).

To better understand the roles of *MaWRKYIIc7* in plants exposed to drought stress, we investigated the changes in the expression of osmotic stress marker genes (including *AtAFP2*, *AtDREB1C*, *AtDREB1D*, *AtABA1*, and *AtARCK1*) (Figure S5). The results revealed that the expression levels of most response genes were significantly upregulated and greater in OEIIc7-2 than in OE1305. Among them, *AtDREB1D* was increased by 2-fold compared with OE1305, *AtABA1* was increased by approximately 3-fold, and *AtAFP2* was also upregulated significantly. In addition, *AtARCK1* and *AtDREB1C* were not activated by *MaWRKYIIc7*.

### 2.6. Transient Overexpression of MaWRKYIIc7 in Mulberry Leaves Enhances Plant Drought Resistance

**Figure 4:**
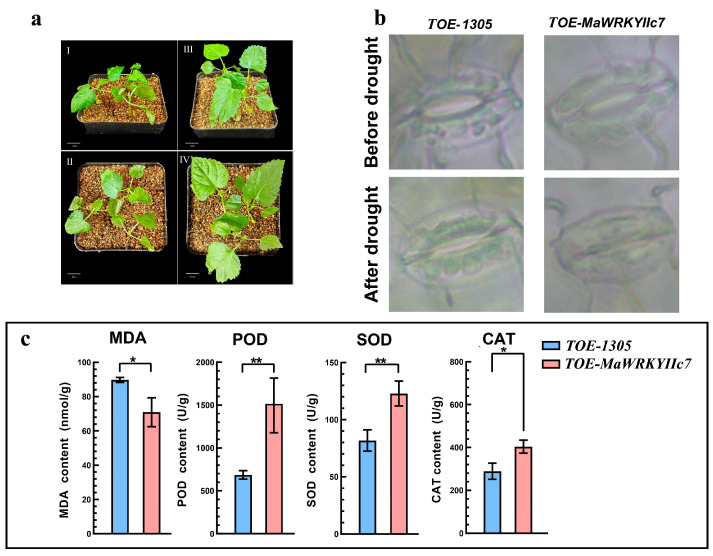
Phenotype of *MaWRKYIIc7* transient overexpression transformed mulberry seedlings after drought treatment. (**a**) Phenotypic analysis of *MaWRKYIIc7* transient overexpression mulberry seedlings after 10 d of drought treatment. I, II image depicts the plants subjected to control treatment (TOE-1305) and III, IV is a genetically modified plant (TOE-*MaWRKYIIc7*). (**b**) Observation of stomatal opening of TOE-1305 and TOE-*MaWRKYIIc7* before and after drought treatment. (**c**) Effects of drought treatment on the contents of MDA, POD, SOD, and CAT in TOE-1305 and TOE-*MaWRKYIIc7* (* significant difference at *p* < 0.05; ** significant difference at *p* < 0.01).

To clarify the biological functions of *MaWRKYIIc7*, we transformed the recombinant plasmids pCAMBIA1305 and pCAMBIA1305-*MaWRKYIIc7* into *Agrobacterium* cells (with transient overexpression in the leaves of mulberry seedlings via the injection method, TOE-*MaWRKYIIc7* and TOE-1305 were obtained). After overexpression, mulberry seedlings began to exhibit a significant atrophy phenotype 10 d after the last watering (Figure 4a). Under drought stress, the stress symptoms of TOE-*MaWRKYIIc7* were significantly lower than those of TOE-1305. For example, the development of mulberry seedling leaves in TOE-1305 was significantly inhibited, with severe shrinkage and curling, and some upright stems were damaged. The leaves of TOE-*MaWRKYIIc7* developed slightly better than those of TOE-1305 did, with some degree of wrinkling, and the middle leaves presented yellowing and curling phenomena. Upon inspection of the stomatal opening of the leaves, TOE-*MaWRKYIIc7* exhibited a significant decrease in the stomatal opening after being subjected to drought stress. Although the degree of stomatal opening in TOE-1305 also decreased, it was still greater than that in TOE-*MaWRKYIIc7* (Figure 4b).

The MDA, POD, SOD, CAT activities (contents) were significantly different between transgenic plants and control group (Figure 4c). The trends of POD, SOD, and CAT activities were similar, with the TOE-*MaWRKYIIc7* content being greater than that of TOE-1305. The MDA content in TOE-1305 was the highest (89.77 nmol/g), whereas that in TOE-*MaWRKYIIc7* was only 70.96 nmol/g. These results indicate that the overexpression of *MaWRKYIIc7* enhances the defence of mulberry seedlings against drought stress, effectively reducing the adverse effects caused by stress.

### 2.7. MaWRKYIIc7 Activates the Expression of Drought Stress-Responsive Genes

**Figure 5:**
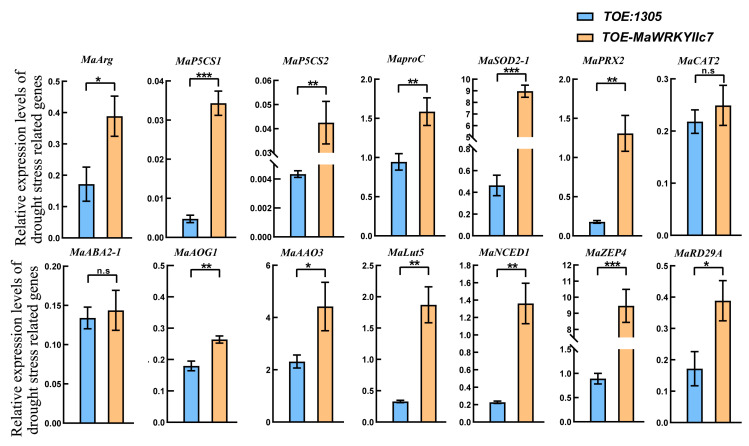
The expression pattern of drought stress related genes in *MaWRKYIIc7* overexpression mulberry (* significant difference at *p* < 0.05, ** significant difference at *p* < 0.01, *** significant difference at *p* < 0.001, n.s indicates no significant difference).

The overexpression of transcription factors may activate (inhibit) multiple genes or pathways. We investigated the changes in the expression levels of several stress-responsive genes related to the drought stress response to better evaluate the roles of *MaWRKYIIc7* in the drought stress response (TOE-1305 as the standard reference) (Figure 5). The expression levels of genes related to proline accumulation (*MaArg*, *MaP5CS1*, *MaP5CS2*, and *MaproC*) and ROS clearance (*MaSOD2-1*, *MaPRX2*, and *MaCAT2*) were greater in TOE-*MaWRKYIIc7* than TOE-1305. The key genes in the ABA synthesis pathway also underwent changes. These results indicate that the overexpression of *MaWRKYIIc7* affects the expression of drought stress responsive genes in mulberry, increasing the drought tolerance of transgenic plants.

**Figure 6:**
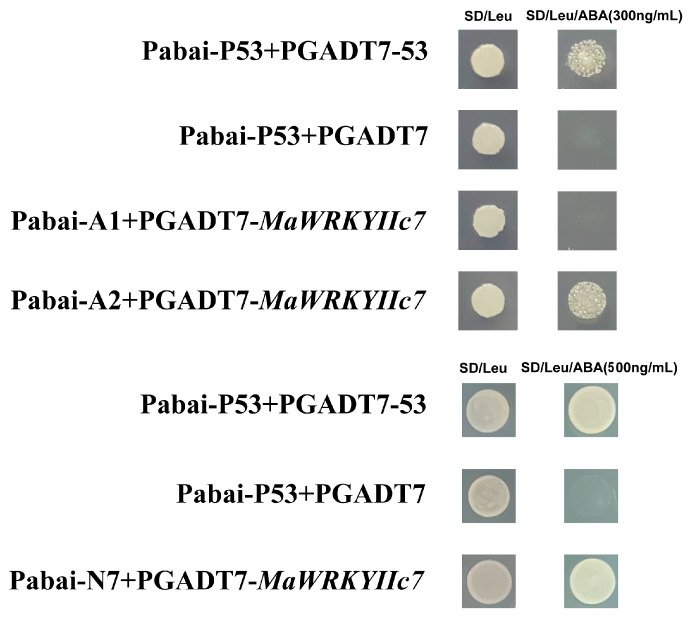
Y1H detection of binding activity between *MaWRKYIIc7* and interactive candidates. A1 and A2 represent fragments of *proMaRD29A*, while N7 belongs to *proMaNCED1*.

**Figure 7:**
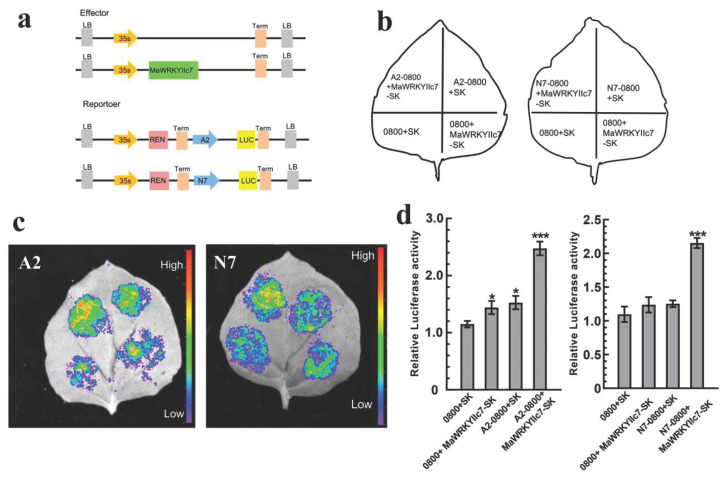
Dual-luc assays validate *MaWRKYIIc7* binding to *proMaRD29A* and *proMaNCED1* to activate their transcription activity. (**a**) Schematic diagrams of the effector and reporter vectors used in Dual-luc assays. (**b**) Layout pattern diagram of tobacco leaf infection. (**c**) Fluorescence intensity measurement of Dual-luc assays. (**d**) Relative luciferase activity detection. Asterisks indicate significant differences (* significant difference at *p* < 0.05, *** significant difference at *p* < 0.001).

Researchers have demonstrated that *WRKY* TFs can regulate plant drought tolerance by modulating the expression of genes such as *RD29A*, *STZ*, and *DREB2* [24]. To investigate the molecular mechanism of *MaWRKYIIc7*, this study revealed that drought stress induced the expression of the mulberry *MaRD29A* (L484_015784) and *MaNCED1* (L484_021647), in which the promoter regions retained *cis*-acting elements related to ABA or stress (ABRE, MBS, and TC-rich repeats) and W-box elements (Figure S6) [23]. On the basis of the different positions of the promoter elements, this study divided the promoters of *MaRD29A* and *MaNCED1* into two segments (A1 and A2) and seven segments (N1-N7), and connected them to the Pabai vector (Table S3). They were subsequently cotransformed into yeast cells with the pGADT7-*MaWRKYIIc7* recombinant vector. The results showed that the yeast combination of Pabai-A2/Pabai-N7 and pGADT7-*MaWRKYIIc7* could grow normally on SD/-Leu/ABA (300 ng/mL or 500 ng/mL) media (Figure 6). Transient transcription assays were then performed to confirm the binding effect of *MaWRKYIIc7* on *MaRD29A*-A2 and *MaNCED1*-N7 via a Dual-luc reporter system (Figure 7a). The *MaRD29A*-A2 and *MaNCED1*-N7 sequences were inserted into the reporter vector to drive the expression of the firefly *luciferase* gene. The *MaRD29A*-A2-0800 and *MaNCED1*-N7-SK recombinant plasmids were transformed into *Agrobacterium*, and the bacterial cells were enriched and cultured to infect tobacco leaves (Figure 7b). The results revealed that the coexpression of *MaRD29A*-A2-Luc and *MaWRKYIIc7* resulted in significantly stronger fluorescence intensity than did the coexpression of *MaRD29A*-A2-Luc and the empty effect vector. Similarly, co-expressing *MaNCED1*-N7-Luc with *MaWRKYIIc7* also resulted in the highest luminescence intensity, indicating that *MaWRKYIIc7* could bind to the promoter regions to activate the transcription of *MaNCED1* (Figure 7c,d). The presence of a W-box in both the A2 and N7 sequences indicates that *MaWRKYIIc7* can bind to the promoters of *MaRD29A* and *MaNCED1* through a W-box.

## 3. Discussion

Drought is an unavoidable topic in agricultural production, which can lead to reduced crop yields [1]. Previous researchers have extensively studied drought resistance genes in plants and analysed the drought stress regulatory network centred on transcription factors such as *MYB*, *AP2/ERF*, and *NAC* [10,25]. *WRKY* TFs constitute one of the largest transcription factor families in plants and are involved in regulating various abiotic stress responses, especially the drought stress response [26,27]. In *Giardia lamblia*, *Dictyostelium discoideum*, *Chlamydomonas reinhardtii,* and other lower species, *WRKY* TFs were identified, indicating that the *WRKY* gene appeared earlier than plant differentiation did [28]. The small size of the *WRKY* gene family in mulberry may be due to large-scale chromosome breakage, duplication, or loss during two major gene replication events in plants [29], which results in some *WRKY* TFs not being retained in the genome.

In this study, we traced 21 duplicated gene pairs in mulberry, all these gene pairs related to duplication events come from the same group, such as *MaWRKYI3*/*MaWRKYI5* and *MaWRKYIIb1*/*MaWRKYIIb3* (Table 1). A study in *Pisum sativum* revealed that 16 *PsWRKYs* involved in 9 tandem duplication events all originated from the same subfamily, but 19 pairs of segmented duplication events involved some combinations of members of different subfamilies [30]. Similar conclusions can also be drawn in *Avena sativa*: 111 *WRKY* TFs duplicated gene pairs are composed of 108 fragment repeats and 3 tandem repeats. Fragment repeats are the main means of amplifying the *Avena sativa WRKY* family [31]. These results indicate that the expansion mechanism of the 111 *WRKY* TFs is different among species and that segmental duplication events may not be the main driving force for the expansion of mulberry *WRKY* TFs. This contrasts with the results of some species with larger transcription factor families, which rely on segmental duplication as a driving force for expansion [32,33,34]. In-depth analysis revealed that other 21 duplicated gene pairs in mulberry, except for *MaWRKYIIe3*-*MaWRKYIIe4* (Ka/Ks = 1.204), underwent strong purifying selection (Table 1). The Ka/Ks values of *MaWRKYIIe3*-*MaWRKYIIe4* are much greater than 1, indicating that under environmental pressure, *MaWRKYIIe3* and *MaWRKYIIe4* have derived new functions after gene duplication events, which is of great significance for the evolution of species [35]. An analysis of the *WRKY* TFs sequence of mulberry revealed that the length range of amino acid sequences was 180–769 amino acids (AA) (Table S1). There were significant differences in kDa values (minimum 20.9, maximum 88.3), which is consistent with the results in *Pisum sativum* (154–710 AA, 17.68–79.12 kDa) [30], indicating that environmental pressure during the evolution of mulberry *WRKY* TFs led to changes in gene structure. We found that approximately 70% of the family members had pI values less than 7.5 (all III group members had values less than 6.5), and most mulberry *WRKY* TFs tended to contain acidic amino acids.

*WRKY* TFs regulate growth and development and secondary metabolism and respond to external pressure in different plant organs. In our study, *WRKY* TFs presented different expression patterns in leaf, fruit, root, stem, staminate flower and pistillate flower, indicating their functional diversity in mulberry (Figure S3). For example, *MaWRKYI5*, *IIa1*, *III1*, *III4*, etc., are highly expressed in fruits and are potential regulatory factors related to fruit development; *MaWRKYI1*, *IIa2*, and *IIe1* are transcriptionally active in flowers and may be involved in regulating the development of flower organs; and *MaWRKYIIe3*, *III4* may play important roles in the morphogenesis of mulberry, as their expression levels in the stem are higher than those in other tissue parts. Unlike other gene families with close evolutionary relationships and similar expression patterns [36], mulberry *WRKY* TFs exhibit significant differences in expression patterns within each subfamily. The peak expression of nine members in group III was distributed in four tissue sites: leaves (4), fruits (1), roots (1), stems (1), and pistil flowers (2). These genes may have undergone functional divergence as a result. As important response factors for plants to cope with abiotic stress, *WRKY* TFs exhibit subfamily-specific involvement in regulation. Each group of the *WRYK* TFs has different biological functions, among which group II may be involved in plant abiotic stress response [37]. For example, *LchiWRKY18*, *EjWRKY17*, and *TaWRKY31* all belong to group II; these genes regulate drought tolerance through various pathways in response to drought stress induction [37,38,39]. We focused our attention on group II mulberry *WRKY* TFs, and found that **6** subfamily members were induced by drought stress, reaching peak expression levels 3–8 d after treatment, which was more than 10 times greater than those in the control group (*MaWRKYIIc2* and *IIc7*) (Figure S3). Notably, none of the six members of IIb were induced by drought stress, indicating that these genes may be involved in the response to other abiotic stresses. Among the *WRKY* TFs in which the expression was induced by drought, *MaWRKYIIc7* expression gradually increased with increasing treatment time, and the expression level in the leaves was significantly greater than that in the other tissues. According to our research as well as those in *Arabidopsis* and *Triricum aestivum*, *MaWRKYIIc7* is closely related to *TaWRKY31* and *AtWRKY57*, and these genes may increase plant drought tolerance [37].

This study further elucidated the biological functions of *MaWRKYIIc7*. We identified ABRE *cis*-acting elements in the *MaWRKYIIc7* promoter region, indicating that this gene may be induced by ABA expression (Table S2). TFs regulate gene transcription inside the nucleus, but extra-nuclear TFs have been found in many TF families, including plant-specific *NAC*, *ARF*, WRKY, etc. These TFs exist in a transcriptionally inactive state outside the nucleus and enter the nucleus through nuclear localisation signals or other pathways when the body is subjected to biotic or abiotic stress [40]. These extra-nuclear TFs can be found in the cytosol or associated with various membrane systems, including the endoplasmic reticulum and plasma membrane. Similarly to the results of soybean (*GmMYB183*) and eggplant (*SmNST1*) [41,42], the recombinant MaWRKYIIc7-eGFP we constructed produced fluorescence signals in both the nucleus and plasma membrane. This result suggests that *MaWRKYIIc7* may be an extranuclear TF that can remain outside the nucleus to control transcriptional activity. However, the mechanisms of *MaWRKYIIc7* nuclear import and export still need further research. (Figure 2a). The transcriptional activation results revealed that full-length *MaWRKYIIc7* and N-terminal segments ranging from 1 to 160 AA grew normally on SD-T/H/A media, whereas C-terminal segments containing conserved WRKY domains (161–310 AA) did not exhibit transcriptional activity (Figure 2b). Research in wheat also showed that conserved WRKY domains are not necessary for the transcriptional activation activity of *WRKY* TFs [25]. We overexpressed *MaWRKYIIc7* in *Arabidopsis* to preliminarily evaluate its biological functions. When mannitol was used to simulate drought stress, as the concentration of mannitol increased, the growth of all the transgenic plants significantly decreased, OEIIc7 seedlings were resistant to stress, the length of the roots of the OEIIc7 seedlings was significantly greater than that of the OE1305 seedlings (Figure 3). Some stress genes related to osmotic stress in *Arabidopsis*, such as *AtDREB1D*, *AtABA1*, and *AtAFP2*, were significantly upregulated in the transgenic lines overexpressing *MaWRKYIIc7* (compared with OE1305) (Figure S5). The overexpression of *MaWRKYIIc7* can increase the drought resistance of overexpressing *Arabidopsis*, and this improved ability to cope with stress may be due to the widespread activation of some related genes. This phenomenon can also be observed in other species, such as *HaWRKY76*, *TaWRKY19*, and *GmWRKY16*, which can confer stronger drought resistance in transgenic *Arabidopsis* via the upregulation of stress and ABA-responsive genes [43,44,45].

We constructed *MaWRKYIIc7* transiently overexpressing mulberry (TOE-*MaWRKYIIc7*) and subjected them to drought treatment under natural conditions (control group transferred to pCAMBIA1305 empty load, TOE-1305) (Figure 4a). After 10 d of natural drought, TOE-*MaWRKYIIc7* presented better drought tolerance than TOE-1305, and the stress symptoms of leaf development damage, atrophy, and curling were also relatively mild. Transcription factors, including *WRKY* TFs, regulate the plant drought stress response, which often relies on the ABA signalling pathway. The ABA signalling pathway regulates the opening and closing of leaf stomata, which can effectively prevent rapid water loss and is an important strategy for plants to cope with drought stress [46]. *Arabidopsis AtWRKY1* can regulate the expression of *MYB2*, *ABCG40*, *DREB1A*, and other ABA signalling pathways, affecting the perception of ABA signals by leaf guard cells to control stomatal opening and closing [47]. *MfWRKY17* has been shown to regulate ABA biosynthesis and stress-related gene expression, controlling stomatal opening and closing to maintain the relative water content [48]. We observed stress phenotypes similar to those associated with the genes mentioned above. Under drought stress, the overexpression of *MaWRKYIIc7* induced stomatal closure in mulberry leaves, resulting in less stomatal opening and water dispersion loss than in TOE-1305 plants (Figure 4b). Moreover, the expression of genes related to the ABA signalling pathway (*Lut5*, *ZEP4*, and *NCED1*) was significantly upregulated in TOE-*MaWRKYIIc7* (Figure 5). These results showed that *MaWRKYIIc7* can activate the ABA signalling pathway in mulberry, regulate leaf stomatal opening and closing, and play a positive role in the plant's response to drought stress. When plants are subjected to abiotic stresses, excessive amounts of ROS accumulate, leading to the oxidation of lipids, proteins, nucleic acids, and even cell death [49]. The MDA content can effectively reflect the degree of lipid peroxidation and is an important indicator for monitoring plant stress capacity [50]. We found that TOE-*MaWRKYIIc7* has greater antioxidant activity than TOE-1305, with higher levels of POD, SOD, and CAT, and a small accumulation of MDA (Figure 4c). The qRT-PCR results confirmed this result, as the expression of ROS scavenging-related genes such as *MaSOD2-1*, *MaPRX2*, and *MaCAT2* was significantly upregulated in TOE-*MaWRKYIIc7* (Figure 5). This finding is consistent with the strategy of activating genes such as *HbWRKY82*, *ZmWRKY40*, and *IbWRKY2* to clear ROS and increase the response of plants to drought stress [51,52,53]. The results of phenotype and physiological indicators measurement showed that the leaves of *MaWRKYIIc7* overexpressing plants had stronger water retention ability and ROS scavenging ability, while the degree of lipid peroxidation in cells was weakened. In summary, *MaWRKYIIc7* can activate ROS scavenging-related genes and regulate stomatal opening and closing through the ABA signalling pathway to increase plant drought tolerance.

Numerous studies have shown that *WRKY* TFs can directly regulate the expression of genes such as *RD29A*, *NCED*, and *ABA3* and can participate in the regulation of the plant drought stress response. *AtWRKY57* and *MaWRKYIIc7* are homologous genes; *AtWRKY57* relies on W-box activation to express *RD26A* and *NCED3*, thereby increasing drought tolerance in *Arabidopsis* [54]. An improvement in wheat drought tolerance can be achieved by combining *TaWRKY19* with the W-box of *DREB2A* and *Cor6.6*, thereby activating *DREB2A*, *RD29A*, *DR29B*, and *Cor6.6* [44]. We identified W-boxes from both *proMaRD29A* and *proMaNCED1*. Y1H and Dual-luc confirm the molecular mechanism of *MaWRKYIIc7*-dependent W-box regulation by *MaRD29A* and *MaNCED1* (Figure 6 and Figure 7). The results of qRP-PCR further confirmed that the upregulation of *MaRD29A* and *MaNCED1* expression was induced by drought and *MaWRKYIIc7* (Figure 5).

On the basis of the above research results, we believe that *MaWRKYIIc7* may interact with *MaRD29A* and *MaNCED1* synergistically and positively regulate the drought tolerance of mulberry through the ABA pathway. Our research enriches the results of plant stress regulation studies, demonstrating the potential of *MaWRKYIIc7* in crop resistance breeding and laying a theoretical foundation for the rapid development of the sericulture industry.

## 4. Materials and Methods

### 4.1. Plant Materials

The mulberry materials used in this study were sourced from the Mulberry Germplasm Resources Nursery (Hefei City, China), and the same water and fertiliser system and management plan were used throughout the entire process. Mulberry seedlings were obtained through seed germination. Drought stress treatment was carried out by controlling irrigation. After the last watering, sampling began when the soil moisture decreased to 25%. The first collection of mulberry seedling materials was recorded at 0 days (d), followed by a continuous collection of drought-treated materials for 3, 5, 8, and 10 d.

### 4.2. WRKY TFs Sequences Bioinformatics Analysis

DNATOOLS1.2.3 software is used to create a local database containing information on mulberry amino acids and nucleic acids. We used the WRKY domain sequence as a search probe and queried the mulberry *WRKY* TFs sequence via the default E value in the TBlastN search. SMART online software (http://smart.embl.de/, 30 January 2025) was used to filter out sequences without the WRKY domain or zinc finger structure, and redundant and repetitive sequences were also removed. The ExPASy website was used to investigate information such as the molecular weights and pI values of *WRKY* TFs (https://www.expasy.org/, 30 January 2025) [55]. The subcellular localisation results were predicted via the WoLF PSORT website tool (https://wolfpsort.hgc.jp/, 30 January 2025) [56]. The *cis*-elements of the *MaWRKYIIc7* promoter were analysed using the Plantcare online website (https://bioinformatics.psb.ugent.be/webtools/plantcare/html/, 30 January 2025).

### 4.3. Ka/Ks Ratio and 4DTV Value Analysis

The appropriate version of OrthoFinder2.0 software was downloaded via Anaconda (https://github.com/davidemms/OrthoFinder/, 30 January 2025), which was built into the backend of the server to identify the orthologous genes of mulberry *WRKY* TFs [57]. The website process can execute the following command: orthofinder-f/input-S diamond-M msa-T fasttree-t 20. The results were analysed via the online tool OrthoVenn3 (https://orthovenn3.bioinfotoolkits.net/, 30 January 2025), and the ClusterVenn module was used to statistically analyse gene clusters and perform sequence alignment of homologous gene pairs via the FFT-NS-i programme of MAFFT7.526 software [58]. The Ka/Ks ratio calculation was performed via the KaKs_Calculator2.0 tool for ParaAT2.0 (parallel alignment and back translation). Simultaneously, the batch_4DTV_calculation script was used to batch calculate the value of 4DTV on the result file processed by ParaAt2.0 [59].

### 4.4. Real-Time Quantitative RT-PCR

Total RNA from different tissues was extracted via TRIzol reagent (Magen Biotechnology, Guangzhou, China). The first strand of cDNA from each sample was obtained via reverse transcriptase (Vazyme, Nanjing, China). The corresponding primers used for RT-PCR are listed in Table S4. Each 20 μL qRT-PCR system consisted of 10 μL of SYBR Premix ExTaq^TM^ II (2×) (Takara, Kusatsu, Japan), 2 μL of cDNA, 6.4 μL of water, and 0.8 μL of upstream and downstream primers. *MaActin* and *MaGAPDH* were used as reference genes, and the relative expression values were calculated (10^−(ΔCt/3)^). Three biological replicates were performed for RT-PCR detection, and the quantitative results are shown as the means ± SDs [60].

### 4.5. Determination of Physiological and Biochemical Indicators Related to Drought Stress

The malondialdehyde (MDA) content and activity of peroxidase (POD), superoxide dismutase (SOD), and catalase (CAT) were determined in 1 g of each leaf sample [23]. MDA determination was carried out using the thiobarbituric acid method. Quartz sand and 0.05 mol/L phosphate buffer were added to a precooled mortar and ground into a homogenate, after which 5 mL of 0.5% thiobarbituric acid solution was added. After shaking well, the mixture was boiled in a boiling water bath for 10 min, cooled, and centrifuged, and the resulting supernatant was taken as the test solution. A 0.5% thiobarbituric acid solution was used as a blank control, and the absorbance of the solution was measured at 532, 600, and 450 nm via a spectrophotometer. The MDA content in the sample was calculated via the formula C = 6.45 (A_532_-A_600_) − 0.56A_450_ and Y = CV/W, where C = MDA concentration (μmol/L), V = extraction volume (mL), W = fresh tissue weight (g), Y = MDA content (nmol/g).

The activity of POD was determined using the guaiacol method. Quartz sand, calcium carbonate, and distilled water were added to the mortar, and the leaf samples were ground thoroughly. The homogenate was transferred to a 50 mL volumetric flask, adjusted to a constant volume, shaken well and centrifuged. The supernatant, which is the POD test solution, was extracted. An amount of 1 mL of the test solution was mixed with 1 mL of 0.1% guaiacol, 6.9 mL of distilled water, and 1 mL of 0.18% hydrogen peroxide. The mixture was shaken well and reacted at 25 °C for 10 min, ensuring the accuracy of the reaction time. To terminate the reaction, 0.2 mL of 5% phosphoric acid was added, the absorbance of the solution was measured at 470 nm, the value on the standard curve was read, and the POD activity in the sample was calculated.

The activity of SOD was determined using the nitro-blue tetrazolium method. Vinylpyrrolidone phosphate buffer was added to the sample in a precooled mortar, which was ground into a homogenate and transferred to a 10 mL volumetric flask and adjusted to a constant volume. The homogenised material was centrifuged, and the supernatant was collected to obtain the crude extract of SOD. A colour reagent was added for the colour reaction, the absorbance of the solution was measured at 560 nm, and the SOD activity in the sample was calculated.

When conducting CAT activity testing, the sample was ground into a homogenate in a phosphate buffer solution and diluted to 10 mL. The mixture was incubated at 4 °C for 10 min and then centrifuged to collect the supernatant, which was the CAT activity test solution. Then, 1.5 mL of phosphate buffer, 1 mL of distilled water, and 0.2 mL of crude enzyme solution were added (the crude enzyme was heated and boiled as a control). The test samples were incubated at 25 °C, and 0.3 mL of hydrogen peroxide (0.1 mol/L) was added. The absorbance was immediately measured at 240 nm, and it was recorded every minute for a total of 5 measurements.

### 4.6. Transformation of MaWRKYIIc7 in Arabidopsis and Mulberry

The pCAMBIA1305-*MaWRKYIIc7* recombinant plasmid was transferred into *Agrobacterium* and enrichment culture (bacterial cells were collected by centrifugation). The infection buffer (0.02% Silwet L-77, 1/2 MS, 5% sucrose) was prepared, and the collected bacterial cells were suspended. A UV spectrophotometer was used to limit the OD_600_ value of the infection buffer to between 0.6 and 0.8 for subsequent infection experiments. When infecting *Arabidopsis*, first, the already grown pods were removed, the flower buds were in full bloom, and fresh flower buds were chosen for infection. During infection, the flower buds that were in good growth condition were soaked for 45 s in the invasion medium, and any remaining infection solution on the flower buds was gently removed after completion. The infected *Arabidopsis* plants were transferred to a dark environment for 1 d of cultivation, ensuring that sufficient water was provided during this period. After being cultivated in the dark, the *Arabidopsis* plants were transferred to a normal environment for cultivation and then infected once every week. During the peak flowering period, a total of three infections were performed. The *Arabidopsis* plants were continuously cultivated, and seeds were collected after they matured.

After the seeds were collected, they were disinfected with 75% alcohol for 1 min and 10% sodium hypochlorite for 13 min. After disinfection, the seeds were rinsed with sterile water four times and then evenly sown on MS solid media supplemented with hygromycin. After alternating between light and dark cultivation for 15 d, the normally growing seedlings were transferred to nutrient soil for further cultivation, and the other seedlings were discarded. The qRT-PCR detection *MaWRKYIIc7* expression levels in positive transgenic plants. The instantaneous transformation of mulberry seedlings is achieved via seedlings that have grown for 15 d after sowing and germination, and the same infection solution as described above was used for infection. A syringe was used to aspirate the infection solution, which was thoroughly injected into the lower epidermis of the mulberry leaves, and the leaves were then incubated in the dark for 36 h.

### 4.7. Subcellular Localization and Transcriptional Activation Analysis

Agrobacterium was enriched and cultured with the pCAMBIA1305-*MaWRKYIIc7* recombinant plasmid and the pCAMBIA1305 empty vector, and the bacterial cells were collected via centrifugation. A tobacco leaf infection solution was prepared (10 mM MES, 10 mM MgCl_2_, and 0.1 mM AS) and this infection solution was used to resuspend the Agrobacterium cells. A UV spectrophotometer was used to set the OD_600_ value of the infection solution between 0.6 and 0.8. Flat tobacco leaves in good condition that had grown for 30 d were selected for infection. A syringe was used to administer the injection solution, which was injected thoroughly into the lower epidermis of the tobacco leaves. The mixture was incubated in the dark for 36 h, ensuring sufficient moisture during this period. After the dark cultivation was complete, the leaf tissue near the injection hole was removed and placed on a glass slide, pure water was added dropwise, the sample was covered with a cover glass, and absorbent paper was used to absorb excess water. After production was complete, the cover glass was placed downwards under a confocal laser microscope to observe the spontaneous fluorescence of eGFP.

The full-length and truncated *MaWRKYIIc7* gene was inserted into the pGBKT7 vector. All of the specific primers used are listed in supplementary Table S2. The various expression vectors, pGBKT7-*MaWRKYIIc7* (1–310 AA), pGBKT7-*MaWRKYIIc7* (1–160 AA), pGBKT7-*MaWRKYIIc7* (161–310 AA), the empty vector pGBKT7 (as a negative vector), and pGBKT7-VP16 (as a positive vector), were transferred into the yeast strain AH109, which was inoculated onto SD/-Trp and SD/-Trp/-His/Ade X-a-Gal plates and cultured for 3 d.

### 4.8. Yeast One-Hybrid Assay

The promoter sequence containing W-box elements was ligated into the pAbAi vector and transformed into yeast Y1H receptor cells after digestion. The minimum AbA concentration was determined, and the recombinant plasmid pGADT7-*MaWRKYIIc7* was translated into Y1H receptor cells containing the abovementioned plasmid [61]. The empty AD plasmid was used as a negative control, and yeast growth was observed on SD/−Leu plates with or without AbA.

### 4.9. Dual-Luciferase Imaging Assays

For the firefly luciferase complementary imaging experiment, the promoter sequence containing the W-box element was inserted into the pGreenII 0800-Luc vector containing the luciferase reporter gene, and the full-length encoding sequence of *MaWRKYIIc7* was inserted into the pGreenII 62-SK vector. The combination of different vectors was infiltrated into tobacco leaves via syringes and cultured in the dark for 2–3 d. After a luciferin (100 μM) spray, the leaves were kept in darkness for 5 min before fluorescence observation. The luciferase and Renilla activities of the infiltrated leaves were measured on a 96-well microplate luminometer (wavelength is 560 nm). The final activity was expressed as the luciferase/Renilla activity ratio [62].

## 5. Conclusions

This study involved evolutionary and expression pattern analysis of the *WRKY* TFs family in mulberry and clarified the molecular mechanism of *MaWRKYIIc7*’s involvement in drought stress response. We believe that the small size of the *WRKY* TFs family in mulberry may be due to a lack of segmental duplication. *MaWRKYIIc7* is induced to express under drought stress and reached its peak expression at 8 d of stress treatment. We created *MaWRKYIIc7* transiently overexpressing lines in mulberry seedlings, and by analysing the changes in stress phenotype, stomatal opening and closing degree, ROS clearance ability, and expression patterns of stress-related genes, we clarified the biological function of *MaWRKYIIc7* in enhancing the plant response to drought stress via the ABA signalling pathway. The Y1H and Dual-luc results indicated that *MaWRKYIIc7* can bind to the W-box, positively regulating *MaNCED1* and *MaRD29A* and synergistically regulating the drought tolerance of mulberry. This study supplements the theoretical research on plant stress regulation and provides a theoretical basis and technical support for the molecular breeding of mulberry. We will use precision breeding methods in the future to create new mulberry varieties with better agronomic traits and apply our research results to production practice.

## Figures and Tables

**Table 1:** Ka/Ks analysis for the duplicated *WRKY* TFs paralogues from mulberry.

Orthologous Gene	Ka	Ks	Ka/Ks
*MaWRKYI3-MaWRKYI5*	0.317	1.432	0.221
*MaWRKYI7-MaWRKYI9*	0.352	1.822	0.193
*MaWRKYIIa1-MaWRKYIIa2*	0.424	2.134	0.199
*MaWRKYIIb1-MaWRKYIIb3*	0.452	1.819	0.249
*MaWRKYIIb1-MaWRKYIIb4*	0.370	1.376	0.269
*MaWRKYIIb2-MaWRKYIIb6*	0.543	2.010	0.270
*MaWRKYIIb3-MaWRKYIIb4*	0.496	1.883	0.263
*MaWRKYIIc2-MaWRKYIIc6*	0.259	1.348	0.192
*MaWRKYIIc3-MaWRKYIIc8*	0.507	1.802	0.281
*MaWRKYIIc10-MaWRKYIIc11*	0.739	1.279	0.577
*MaWRKYIIe3-MaWRKYIIe4*	0.625	0.519	1.204
*MaWRKYIIe3-MaWRKYIIe5*	0.541	2.103	0.257
*MaWRKYIIe4-MaWRKYIIe5*	0.641	0.843	0.761
*MaWRKYIII1-MaWRKYIII4*	0.485	1.673	0.290
*MaWRKYIII1-MaWRKYIII8*	0.417	1.668	0.250
*MaWRKYIII2-MaWRKYIII3*	0.138	0.239	0.578
*MaWRKYIII2-MaWRKYIII6*	0.732	1.994	0.367
*MaWRKYIII3-MaWRKYIII5*	0.138	0.239	0.578
*MaWRKYIII3-MaWRKYIII6*	0.756	2.042	0.370
*MaWRKYIII4-MaWRKYIII8*	0.432	1.400	0.309
*MaWRKYIII5-MaWRKYIII6*	0.732	1.994	0.367

## Data Availability

The following information was supplied regarding data availability: the mulberry genome can be found in the Mulberry Genome Database (https://morus.biodb.org/links, accessed on 30 January 2025). The following information is available at https://phytozome-next.jgi.doe.gov/, 30 January 2025: *Arabidopsis*, *Eucalyptus grandis*, *Brachypodium distachyon*, and *Medicago sativa*.

## Supplementary Materials

The following supporting information can be downloaded at https://www.mdpi.com/article/10.3390/ijms26041714/s1.
