# Supplementary material for: The Mulberry WRKY Transcription Factor MaWRKYIIc7 Participates in Regulating Plant Drought Stress Tolerance
Source: Int J Mol Sci. 2025 Feb 17;26(4):1714. doi: 10.3390/ijms26041714 (PMC11855790; doi:10.3390/ijms26041714)
Supplement: Supplementary file 1 [file ijms-26-01714-s001.zip › Table S1.pdf]

Table S1. Statistical analysis of basic information of *WRKY* TFs family members in mulberry.

Gene name	Genome number	AA number	Molecular weight (kDa)	pI	GRAVY value	Subcellular Localization
MaWRKYI1	L484_020026	349	38.8	9.14	-1.042	nucl
MaWRKYI2	L484_015130	742	80.9	6.16	-0.624	nucl
MaWRKYI3	L484_015723	540	58.3	6.33	-0.892	nucl
MaWRKYI4	L484_003860	484	53.8	8.98	-0.974	nucl
MaWRKYI5	L484_020954	495	53.5	8.19	-0.804	nucl
MaWRKYI6	L484_005270	743	79.5	5.73	-0.643	nucl
MaWRKYI7	L484_014007	531	59.2	7.22	-0.987	nucl
MaWRKYI8	L484_009509	571	62.2	6.82	-0.775	nucl
MaWRKYI9	L484_011135	559	61.3	6.71	-0.922	nucl
MaWRKYIIa1	L484_013515	312	34.8	8.66	-0.766	nucl
MaWRKYIIa2	L484_013217	330	36.8	7.60	-0.581	nucl
MaWRKYIIa3	L484_013216	286	32.1	8.93	-0.963	nucl
MaWRKYIIb1	L484_014181	619	66.7	5.87	-0.663	nucl
MaWRKYIIb2	L484_021097	604	66.1	6.02	-0.815	nucl
MaWRKYIIb3	L484_008065	585	63.5	6.06	-0.636	nucl
MaWRKYIIb4	L484_011928	600	65.2	6.99	-0.739	nucl
MaWRKYIIb5	L484_001997	536	58.3	6.06	-0.628	nucl
MaWRKYIIb6	L484_024154	769	88.3	5.22	-1.178	nucl
MaWRKYIIb7	L484_007627	645	70.0	6.43	-0.794	nucl
MaWRKYIIc1	L484_010632	421	46.4	6.23	-0.936	nucl
MaWRKYIIc2	L484_017459	202	22.8	9.33	-0.837	nucl
MaWRKYIIc3	L484_003782	192	22.0	5.57	-1.156	nucl
MaWRKYIIc4	L484_001635	240	26.7	6.82	-0.778	Chlo or nucl
MaWRKYIIc5	L484_021488	212	23.7	9.61	-0.804	nucl
MaWRKYIIc6	L484_010795	180	20.9	9.13	-1.184	nucl
MaWRKYIIc7	L484_006651	310	33.4	5.24	-0.749	nucl
MaWRKYIIc8	L484_008323	210	23.3	7.05	-0.921	nucl
MaWRKYIIc9	L484_009437	316	35.4	4.93	-0.846	nucl
MaWRKYIIc10	L484_002784	433	48.5	6.51	-0.924	nucl
MaWRKYIIc11	L484_021378	380	42.3	6.24	-0.933	nucl
MaWRKYIIc12	L484_005172	257	29.1	8.20	-0.790	nucl
MaWRKYIIc13	L484_010126	186	21.6	9.38	-1.149	nucl
MaWRKYIId1	L484_005757	352	39.2	9.64	-0.744	nucl
MaWRKYIId2	L484_007236	326	35.1	9.52	-0.525	nucl
MaWRKYIId3	L484_000558	366	39.9	9.34	-0.643	nucl
MaWRKYIId4	L484_019849	368	39.3	9.68	-0.508	nucl
MaWRKYIId5	L484_019036	306	34.2	9.56	-0.646	nucl
MaWRKYIIe1	L484_025955	502	53.8	5.86	-0.704	nucl
MaWRKYIIe2	L484_009739	268	30.5	5.62	-0.874	nucl
MaWRKYIIe3	L484_012914	361	38.8	6.32	-0.621	nucl

MaWRKYIIe4	L484_006704	330	37.4	5.78	-0.750	nucl
MaWRKYIIe5	L484_014218	429	47.4	5.68	-0.869	nucl
MaWRKYIIe6	L484_021355	258	27.8	5.35	-0.588	nucl
MaWRKYIIe7	L484_009921	511	56.7	5.58	-0.996	nucl
MaWRKYIIe8	L484_000931	298	33.0	5.49	-0.726	nucl
MaWRKYIII1	L484_011458	405	44.9	5.96	-0.804	nucl
MaWRKYIII2	L484_004213	332	37.0	6.10	-0.654	nucl
MaWRKYIII3	L484_013973	331	37.6	5.55	-0.566	nucl
MaWRKYIII4	L484_017596	364	40.6	5.30	-0.740	nucl
MaWRKYIII5	L484_013971	329	37.6	6.09	-0.562	nucl
MaWRKYIII6	L484_011259	360	41.0	5.09	-1.109	nucl
MaWRKYIII7	L484_011258	346	38.1	5.63	-0.647	nucl
MaWRKYIII8	L484_012050	348	38.8	5.26	-0.697	nucl
MaWRKYIII9	L484_013969	332	37.1	6.22	-0.675	nucl
