# Supplementary material for: The Mulberry WRKY Transcription Factor MaWRKYIIc7 Participates in Regulating Plant Drought Stress Tolerance
Source: Int J Mol Sci. 2025 Feb 17;26(4):1714. doi: 10.3390/ijms26041714 (PMC11855790; doi:10.3390/ijms26041714)
Supplement: Supplementary file 1 [file ijms-26-01714-s001.zip › Table S2.pdf]

Table S2. Analysis of cis-element of *MaWRKYIIc7* promoter.

Elements	Number	Function
ABRE	1	cis-acting element involved in the abscisic acid responsiveness
G-box	1	cis-acting regulatory element involved in light responsiveness
GT1-motif	1	light responsive element
MBS	1	MYB binding site involved in drought-inducibility
ARE	4	cis-acting regulatory element essential for the anaerobic induction
I-box	1	part of a light responsive element
CGTCA-motif	2	cis-acting regulatory element involved in the MeJA-responsiveness
TGACG-motif	2	cis-acting regulatory element involved in the MeJA-responsiveness
