# Supplementary figures and images for: The Mulberry WRKY Transcription Factor MaWRKYIIc7 Participates in Regulating Plant Drought Stress Tolerance
Source: Int J Mol Sci. 2025 Feb 17;26(4):1714. doi: 10.3390/ijms26041714 (PMC11855790; doi:10.3390/ijms26041714)

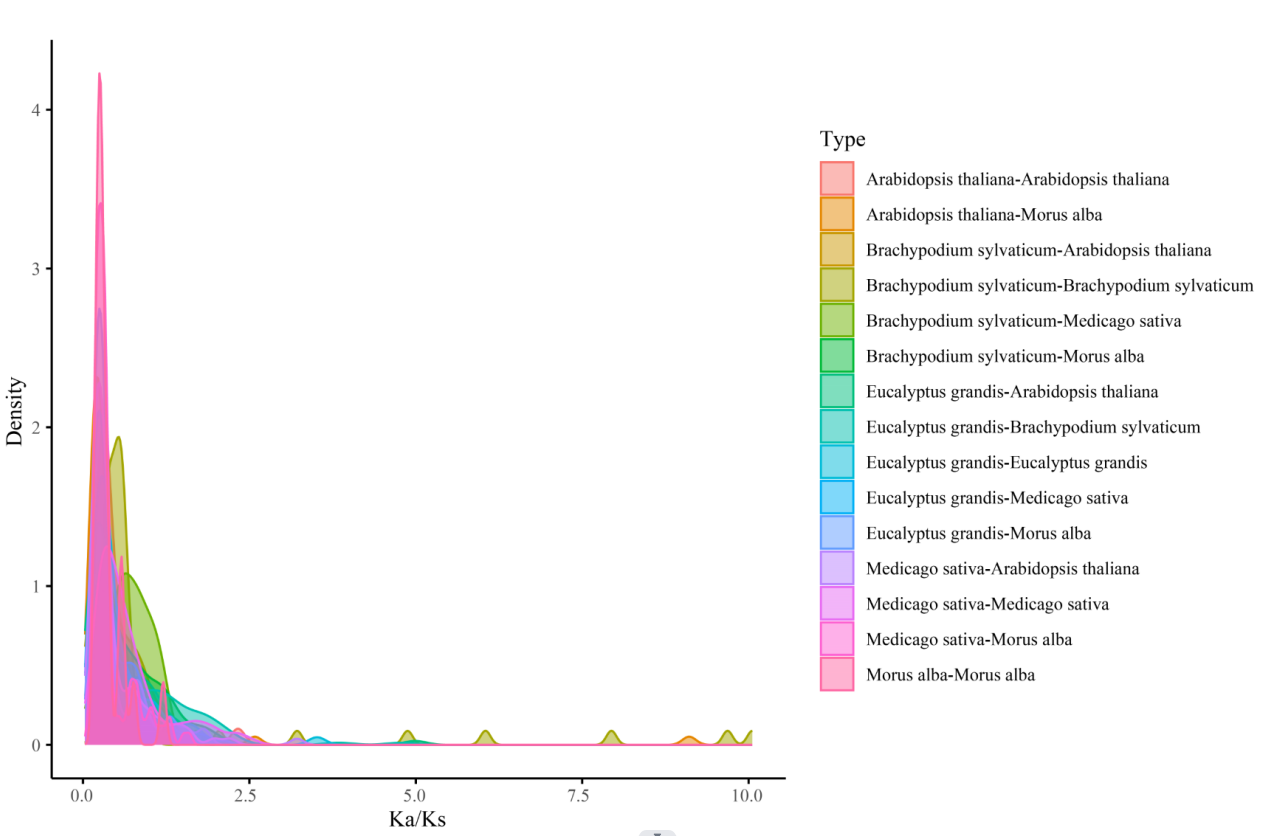

Supplement: Supplementary file 1 [file ijms-26-01714-s001.zip › Figure S1.tif]

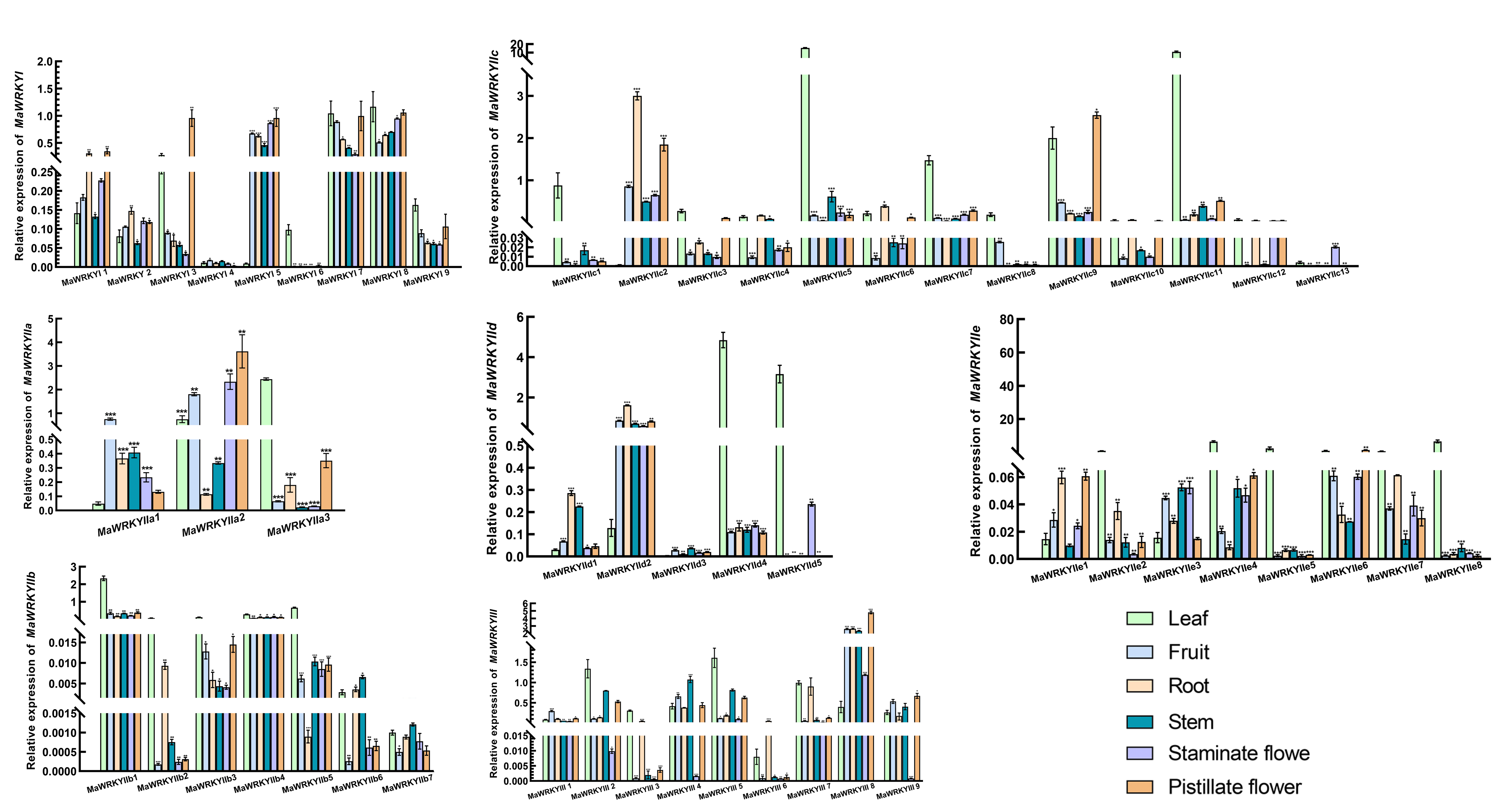

Supplement: Supplementary file 1 [file ijms-26-01714-s001.zip › Figure S2.tif]

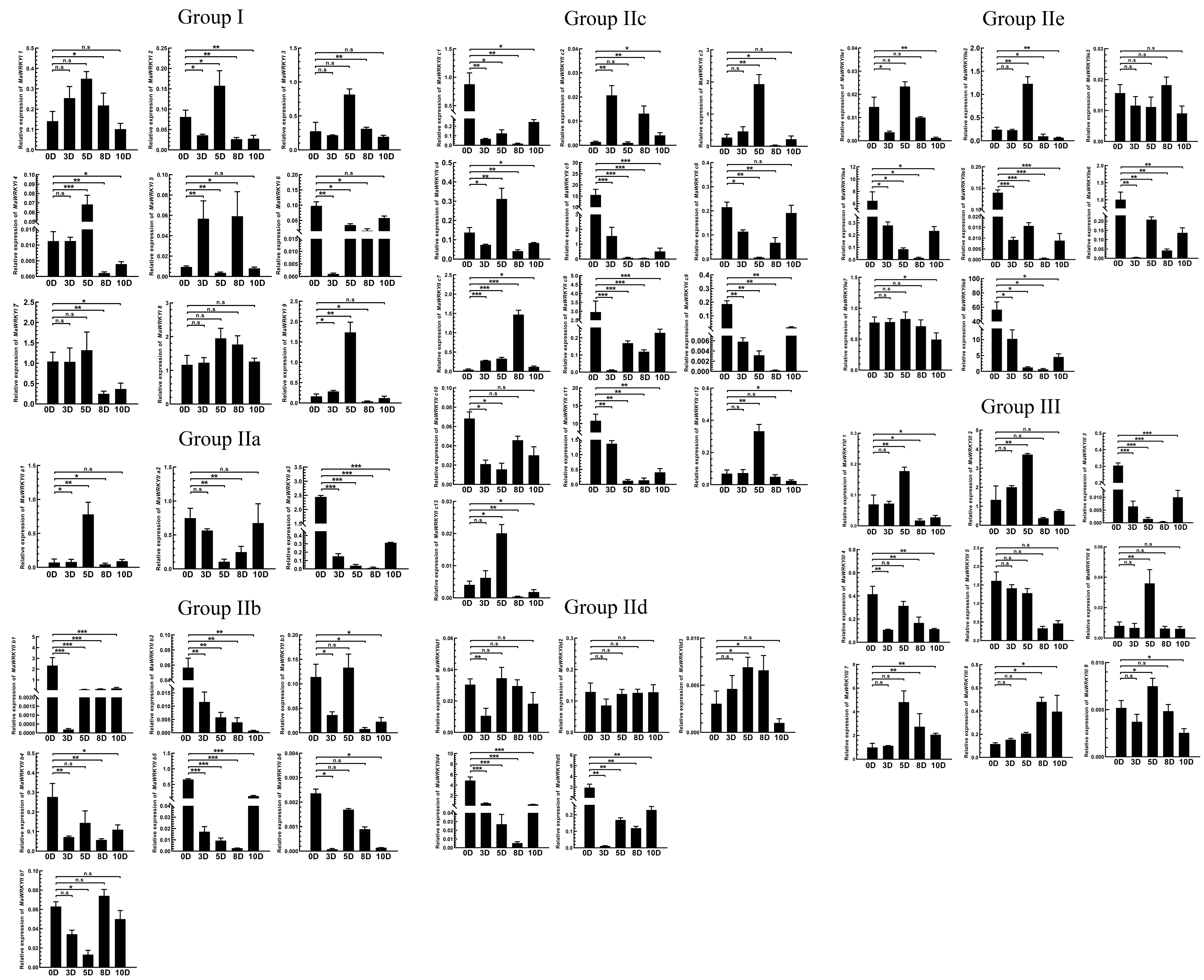

Supplement: Supplementary file 1 [file ijms-26-01714-s001.zip › Figure S3.tif]

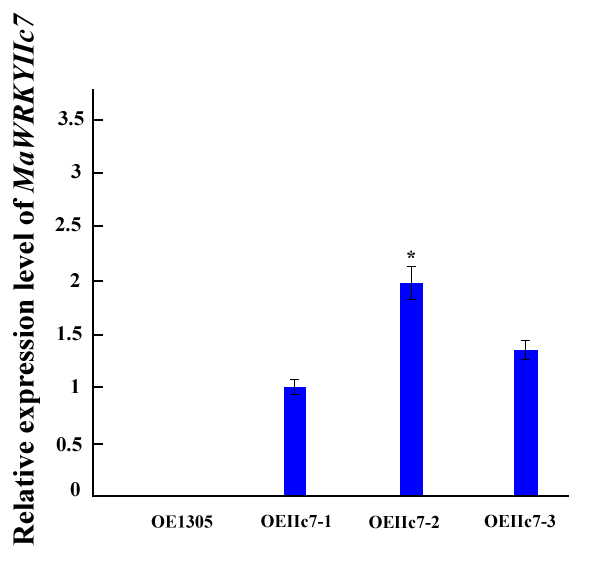

Supplement: Supplementary file 1 [file ijms-26-01714-s001.zip › Figure S4.tif]

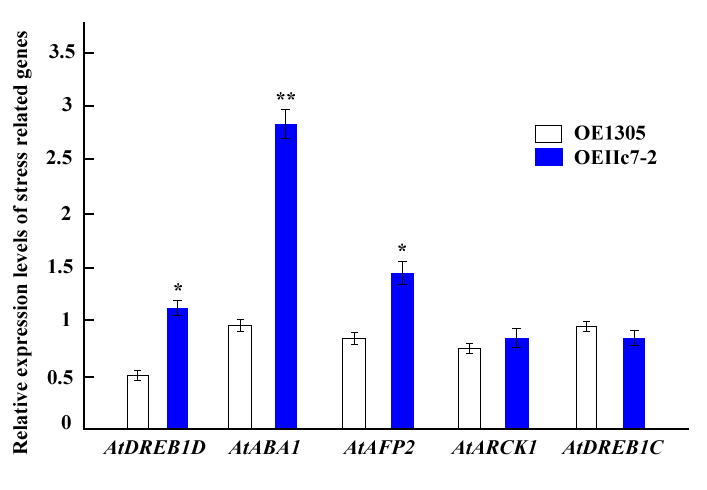

Supplement: Supplementary file 1 [file ijms-26-01714-s001.zip › Figure S5.tif]

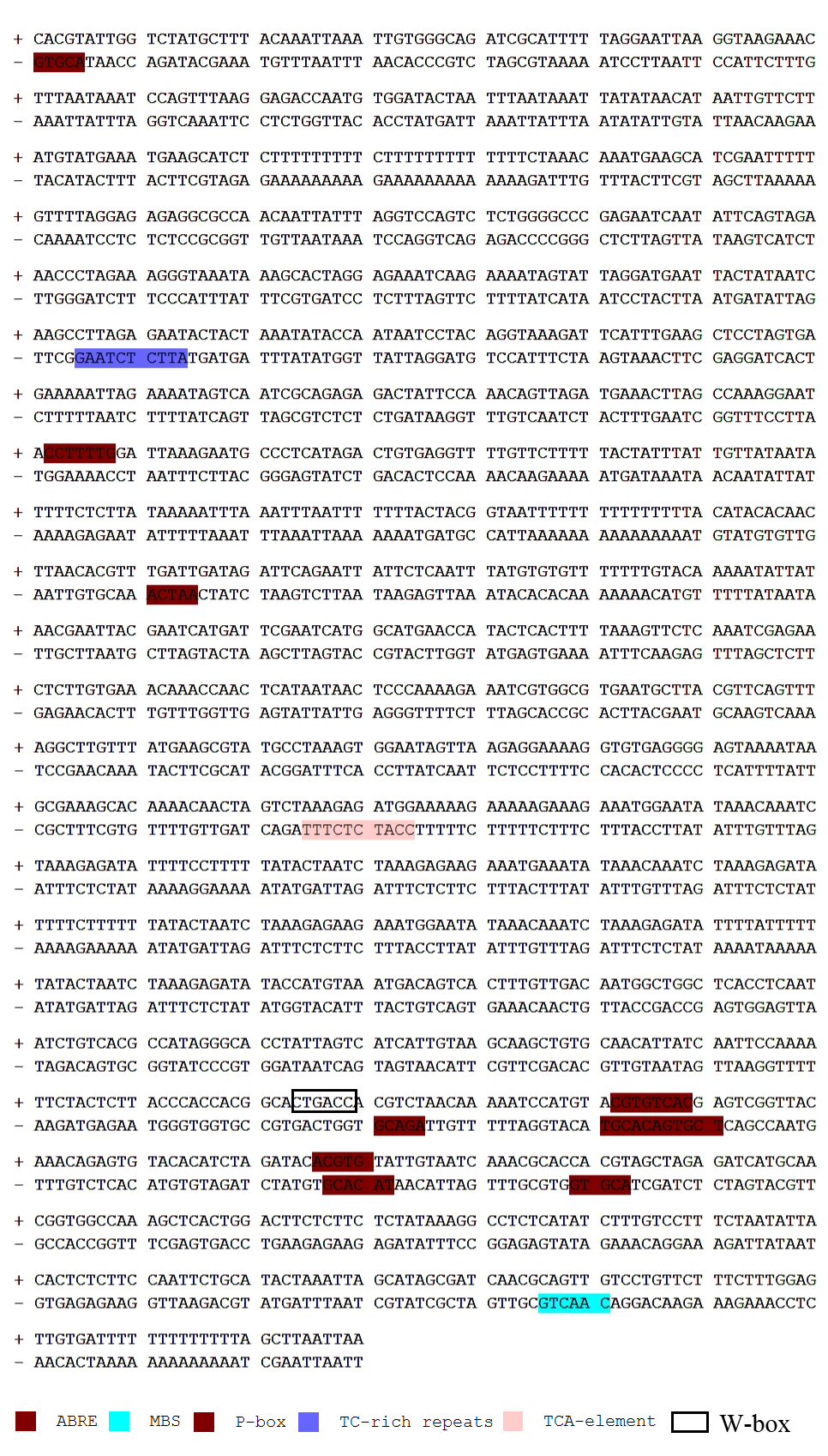

Supplement: Supplementary file 1 [file ijms-26-01714-s001.zip › Figure S6.tif]
